# Reproducibility and replicability in research: What 452 professors think in Universities across the USA and India

**DOI:** 10.1371/journal.pone.0319334

**Published:** 2025-03-26

**Authors:** Tatiana Chakravorti, Sai Koneru, Sarah Rajtmajer

**Affiliations:** 1 Information Science and Technology, Pennsylvania State University, State College, Pennsylvania, United States of America; University of Rennes 1, FRANCE

## Abstract

In the past decade, open science and science of science communities have initiated innovative efforts to address concerns about the reproducibility and replicability of published scientific research. In some respects, these efforts have been successful, yet there are still many pockets of researchers with little to no familiarity with these concerns, subsequent responses, or best practices for engaging in reproducible, replicable, and reliable scholarship. In this study, we surveyed 452 professors from universities across the USA and India to understand perspectives on scientific processes and identify key points for intervention. Our findings reveal both national and disciplinary gaps in attention to reproducibility and transparency in science, aggravated by incentive misalignment and resource constraints. We suggest that solutions addressing scientific integrity should be culturally-centered, where definitions of culture should include both regional and domain-specific elements. This study examines research cultures in India and the USA across a diverse range of social science and engineering disciplines. The universities included in the study were carefully selected to represent various regions of each country and reflect institutions across different ranking levels, ensuring a broad and representative sample. While the findings provide valuable insights into the research environments of India and the USA, their applicability is limited to these two countries and respective disciplines. The survey relies on self-reported data, which can be subject to biases, e.g., social desirability or recall bias. Future research will expand the scope to include additional countries, allowing for a more comprehensive comparison of global research cultures. Additionally, we aim to investigate how regional, institutional, and disciplinary factors influence research practices and collaboration across borders, providing a deeper understanding of international academic environments.

## Introduction

Reproducibility and replicability have gained significant attention in scientific discourse, deeply intertwined with questions about scientific processes, policies and incentives [1–5]. There has been some ambiguity around these terms; we adopt definitions from [5–7]. Reproducibility refers to computational repeatability – obtaining consistent computational results using the same data, methods, code, and conditions of analysis; replicability means obtaining consistent results on a new dataset using similar methods. Initially centered around the social and behavioral sciences, these concerns now span almost all empirical scientific disciplines [8], including artificial intelligence and machine learning [9,10]. The open science and science of science communities have responded with innovative initiatives aimed at shoring up the entire research workflow, from conception and study design to data collection and analysis, through to publishing and [11–13]. These efforts have already had important individual and institutional impacts, many of which have been well-documented [14,15]. For example, the Special Interest Group on Computer-Human Interaction (SIGCHI) now recommends providing supplementary materials for ACM publications to enhance replicability [16] and some universities have begun to reward researchers whose work aligns with standards of open science and transparency [17].

Despite these promising advances, however, conversations around reproducibility and replicability have predominantly reflected the voices of researchers in the global North and West [18–21]. This is concerning for a number of reasons, most primarily because issues of scientific integrity and scientific process are deeply social and contextual. Our work takes an initial step toward the inclusion of cultural perspectives through a comparative study of researchers in the USA and India. India currently ranks third in research output worldwide, following China and the USA [22].

We conduct a survey-based study involving faculties from all different levels of their career for example assistant professors, associate professors, and professors from universities in the USA and India. We aim to gather the perspectives of scientists across different research disciplines and across cultures. Our survey asks participants about their familiarity with the reproducibility crisis, their confidence in work published within their fields, and the factors they believe contribute to this high or low-confidence research. Additionally, we asked participants to share the institutional and practical challenges they faced during their research. We reached out to over 8700 research faculty members and received a total of 452 responses. We ask:

- **RQ1**: What are researchers’ **experiences** around reproducibility, replicability, and open science? How do these experiences differ across culture and domain?
- **RQ2**: How do **institutional factors** contribute to reproducibility and replicability, or lack thereof? How do these factors differ across culture and domain?

Our findings contribute to the global conversation on scientific integrity, underscoring the need to understand challenges and solutions in cultural context. Our findings highlight a number of biases and compound inequalities which have not been fully appreciated by the open science community. We provide recommendations for the stakeholders across the scientific landscape.

## Related work

Our work contributes to the existing literature on scientific practice, particularly reproducibility and open science.

### The reproducibility ‘Crisis’

Attention to reproducibility and replicability have intensified over the past decade thanks to a number of high-profile findings. Large-scale replication projects in psychology [23], economics [24], sociology [25], biology [26] and beyond have turned up disappointing results. In 2016, a survey published in Nature reported that more than 70% of researchers have attempted and failed to reproduce other scientists’ experiments, and more than half have been unable to reproduce their own [8]. The same paper reported that 52% of surveyed researchers believe that there is a significant ‘crisis’ of reproducibility in science. However, the paper fails to collect lots of data from countries like India and continents like Africa.

Initially centered around the social and behavioral sciences, concerns about reproducibility and replicability now span almost all empirical disciplines [8] including artificial intelligence and machine learning [9,10]. Scholars have pointed to a number of reasons for the crisis. These include questionable research practices, such as p-hacking and HARKing (hypothesizing after results are known) [27], selective analysis, selective reporting [28], and lack of transparency [29]. Other contributors to low replication rates include misaligned incentives and failures of peer review [30–32]. These factors diminish researchers’ motivation to conduct quality checks, prompting them to prioritize publishability over reliability.

### Open science

Concerns about reproducibility and replicability are closely related to principles and practices of open science [33–36]. The UNESCO Recommendation on Open Science defines open science, sweepingly, as “an inclusive construct that combines various movements and practices aiming to make multilingual scientific knowledge openly available, accessible and reusable for everyone, to increase scientific collaborations and sharing of information for the benefits of science and society, and to open the processes of scientific knowledge creation, evaluation, and communication to societal actors beyond the traditional scientific community. It comprises all scientific disciplines and aspects of scholarly practices, including basic and applied sciences, natural and social sciences, and the humanities, and it builds on the following key pillars: open scientific knowledge, open science infrastructures, science communication, open engagement of societal actors and open dialogue with other knowledge systems [37]. In this context, researchers have begun to scaffold clear and specific practices that align with open science; chief amongst them is the notion of transparency. Transparent research practices include sharing data and code, comprehensive detailing of methodologies, and clear identification of theoretical foundations [11,38]. Researchers have found that making code available has a positive correlation with increased citations [39]. The specific character of best practices, of course, varies across disciplines [40]. Many fields are working to establish their own norms inspired by open science ideals [41–43] but they have their own challenges.

Over the last two decades, the operational procedures of scholarly social science have been substantially modified to facilitate the goals of open science [11,44,45]. Many journals and conferences in diverse disciplines have begun to adopt reproducibility standards. The literature has also considered engagement in open science through the lens of behavioral change theory (e.g, [46]) and explored ways to enhance the adoption of open science practices among researchers [47,48] and journal editors [49].

Yet, open science practices are still not mainstream. For instance, Gunzer et al. [50] analyzed 83 articles on AI neuroimaging models published between 2000 and 2020, finding that only 10.15% included open-source code. Similarly, a recent survey from the ACM Conference on Learning Scale revealed that none of the 93 papers from 2021-2022 had a corresponding preregistration, and only one used a dataset that was made openly available [51]. In a study examining open science norms in clinical psychology, while 98% of 100 papers sampled between 2000 and 2020 had some data available, only one provided an analysis script [52].

## Methodology

We take an exploratory, survey-based approach for a comparative analysis of researchers’ perspectives on reproducibility, replicability, transparency, and open science in India and the USA. We have followed the CHERRIES (Checklist for Reporting Results of Internet E-Surveys) guidelines and mentioned all required items in our Methods section. All recruitment materials, survey instruments, and anonymized survey responses are publicly available on the paper’s Github repository: https://github.com/Tatianachakravorti/ReproducibilitySurveyData.

### Survey recruitment

We selected 25 universities randomly from the top 100 universities in India based on the National Institutional Ranking Framework (NIRF) [53]. We have maintained the diversity of the location during the selection of these universities so that the responses should not come from a certain location. Likewise, we selected 25 universities randomly from the top 100 in the USA based on the 2023 US News and World Report rankings [54].

We directly emailed research faculty listed on departmental web pages using email addresses collected via web scraping of Universities’ directories. Prior works [55,56] have previously employed this process to identify high-quality participants. We targeted the following disciplines in the social sciences: economics, political science, education, psychology, sociology, and marketing. From engineering, we targeted: computer science engineering; electrical engineering; electronics engineering (India); and mechanical engineering. In total, we emailed 4300 faculty members in India (1268 social sciences, 3032 engineering) and 4400 in the USA (2100 social sciences, 2300 engineering). We sent the survey to professors at all career stages – assistant, associate, and full. We have made this more clear in the current version of the paper. In this study, we did not include PhD students and postdoctoral fellows, however, we appreciate that this population is important and adds valuable perspectives. Our email contained a link to the survey, deployed as a Google Form. A one-time follow-up email was sent to all recipients approximately two weeks later. Participants with automated vacation responses were emailed upon their return. Participation was voluntary. A total of 452 respondents completed the survey, 191 from India (45 social science, 146 engineering) and 261 from the USA (189 social science, 72 engineering). This represents a 4.44% response rate from India and 5.93% from the USA.

### Data collection

Our survey protocol included 20 questions, where four questions are demographic. One question was regarding their academic position. We have deleted that response from the survey to protect participants’ privacy. (3 demographic + 1 academic position + 16 other questions (11 other closed- and 5 open-ended questions)). We asked participants to share their perceptions of the state of reproducibility and open science in their respective academic communities and disciplines, factors they believe contribute to the lack of reproducibility and replicability of findings, challenges, and opportunities to promote reproducible research practices. The survey has been built based on the previous survey design techniques [8,57,58]. In this study, our initial intent was to protect the privacy of participants. Our purpose was only to extract knowledge about the countries where they work and the research area. Therefore we have not considered taking some of the demographic questions for example their age and gender. In this survey, we have asked researchers about their research area, academic position, their country of work, and their country of residence. We have deleted the details of the academic positions while sharing the data to maintain greater privacy. A pilot version of the survey was created and pretested before deployment. The final survey took approximately 15 minutes to complete. The survey was a combination of open-ended and closed-ended questions. Our complete survey instrument is available on the paper’s GitHub repository: https:// github.com/Tatianachakravorti/ReproducibilitySurveyData.

### Data analysis

In this survey, there were two different types of questions, open-ended and closed-ended. Therefore two methods have been used to analyze the data, descriptive statistics, and content analysis. Survey responses for closed-ended questions were analyzed using descriptive statistics [59] and exploratory data visualizations. Open-ended questions (free text responses) were analyzed using content analysis [60,61]. This qualitative data analysis approach uses a thorough examination of free-text responses to identify and quantify patterns related to the research questions. In this study, the first two authors examined all open-ended responses to focus on the manifest content relevant to the survey question and establish initial codes. After that, codes were organized into categories based on similarities and relationships. Lastly, we refined these categories, assigned names to each theme, and crafted a conceptual framework to address our survey questions with the whole team. Our complete data analysis is available on the paper’s GitHub repository: https://github.com/Tatianachakravorti/ReproducibilitySurveyData.

### Ethical statement

Our study directly addresses research ethics and transparency. An explicit aim of our work is to seed more inclusive conversations around reproducibility, research integrity, and highlight perspectives of researchers outside the Western context who we argue have historically been marginalized in studies on this topic. The survey was not preregistered. While we followed a detailed protocol and have tried so that there is no selection bias, preregistration might have strengthened the study’s methodological transparency and rigor.

The study plan received an ethics waiver approved by the Pennsylvania State University’s Institutional Review Board (IRB) before starting data collection. The study number is STUDY00023920. Participants were fully informed about the nature of the study, potential risks, and their right to withdraw at any time without penalty before the study began. Consent was obtained before taking the survey questions explicitly mentioned in the IRB. We have only considered the responses where the participants have provided consent for the survey. Data was stored securely and only used for agreed-upon purposes.

## Findings

This section describes all the quantitative and qualitative findings from the survey responses collected and is divided into sections according to the survey questions.

### Awareness and concern about reproducibility and replicability

**Fig 1:**
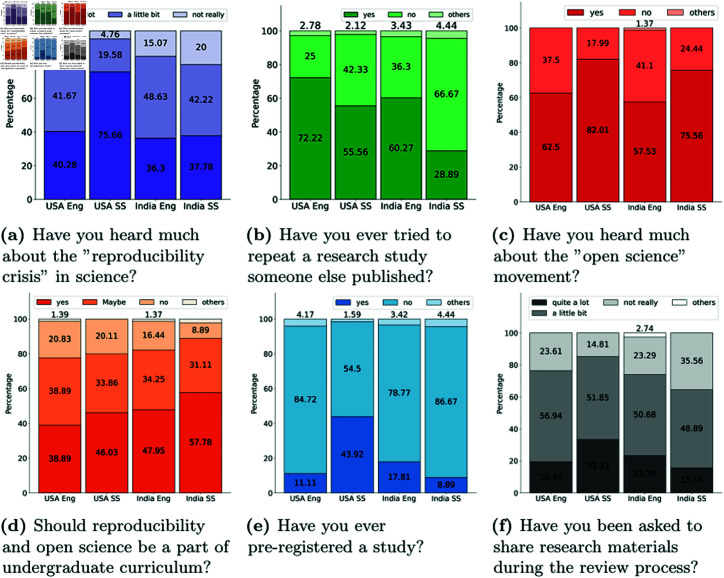
Survey responses by country and domain (Eng = engineering; SS = social science).

This section describes the quantitative findings from the two survey questions which ask them regarding their familiarity with the “reproducibility crisis” and to what extent they think their peers (colleagues in your field) are concerned about the “reproducibility crisis”. We have used descriptive statistics to analyze the findings. **Approximately 83.8% of surveyed researchers in India indicated some level of familiarity with the reproducibility crisis in science. In the USA, awareness was over 91.5%.** Breaking these totals down further, we observe appreciable differences between disciplines. More than 95.23% of social science researchers in the USA are aware of the reproducibility crisis vs. 81.95% in engineering. While, in India, this gap is smaller, with 84.93% of researchers in engineering and 80% in the social sciences endorsing awareness of these concerns (see Fig 1a). Additionally, we explored the respondents’ perceptions of their peers’ awareness of the crisis. **We find that 26.17% of participants from India and 17.62% from the USA believe their peers to be completely *un*aware of the replication crisis.** These statistics underscore differences in open discussions about scientific credibility and practice in India and the USA.

### Factors contributing to lack of reproducibility

Participants were asked about the factors contributing to the lack of reproducibility in their fields. In response, 59.58% of Indian engineering researchers and 65.27% of US engineering researchers identified the unavailability of raw data as a primary obstacle. The unavailability of code was another significant reason for engineering researchers; 58.33% of engineers from the USA and 53.42% from India mentioned it as a challenge. Selective reporting was a concern for 67.72% of US social science researchers and 53.33% of Indian social science researchers.

Publication pressure was acknowledged by 57.85% of respondents from the USA and 45.94% from India. This total was even higher in the social sciences; 62.43% social science researchers from the USA selected publication pressure as a significant contributor to the reproducibility crisis. Insufficient peer review was mentioned by 30.54% of Indian researchers, compared to 21.26% from the USA. Further detail is provided in Fig 3.

**Fig 2:**
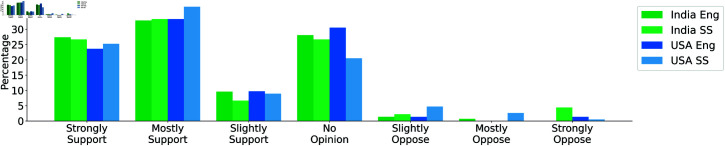
Opinions about the open science movement.

**Fig 3:**
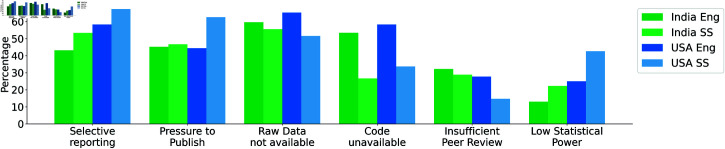
Factors contributing to lack of reproducibility.

For this survey question “other option” was provided so that participants could provide any other challenges not mentioned in the closed options in the question. A variety of other factors came up in these free responses. These included a lack of diversity in the sample population, lack of reliable data, sloppy work/fraud, declining moral standards, privacy issues, industry boundaries, no funding, and constant demand for novel research. A participant from the USA mentioned that the skill of the replicators is also another factor to be considered.

*skill of the replicator: people just don’t know what they are doing. So some wet-behind-the-ears graduate student takes it upon themselves to try and replicate something and miss codes the variables. Could open code fix that? Sure, but we’re talking about missing value code-level mistakes. People just need to learn how to code.* – respondent from USA, sociology

One participant noted that hardware studies are difficult to reproduce because of the high cost.

*Intrinsic difficulties in producing the hardware required to perform tests, and the intrinsic high cost of performing human biomechanics research studies. And: who is going to fund this?? Granting agencies rarely fund new work we propose, let alone propose to repeat someone else’s iffy work.* – respondent from USA, mechanical engineering

It was also noted that qualitative research is hard to reproduce.

*Qualitative research is hard to “reproduce” given the time constraints associated with its inception. Thus, it seems out of balance to focus on one type of study to “reproduce” ignoring a large type of work. This would seem to further divisions between quant and qual work that the discipline has sought to lessen over the years.* – respondent from USA, sociology

### Experiences reproducing and replicating others’ findings

We asked researchers if they ever tried to repeat a research study someone else published using one closed-ended question and asked them to describe their experiences about the replication with an open-ended question. When asked about their experiences replicating others’ work, many researchers noted that they repeat others’ experiments before extending them in their own studies. **52.87% of respondents in India and 60.15% in the USA indicated that they had tried to replicate others’ work.**

Researchers who reported having engaged in replication attempts were asked to share insights about their experiences as an open-ended question. Only 14.58% of Indian researchers who reported trying to replicate others’ research obtained affirmative results with the remaining majority reporting unsuccessful or only partially successful results. In comparison, 33.96% of respondents in the USA who engaged in replication of others’ work reported affirmative results. Looking at disciplinary impacts, we find that 55.55% of social science researchers in the USA have attempted to replicate others’ research vs. 28.89% in India (see Fig 1(b)). In engineering disciplines, 72.22% of participants from the USA and 60.27% from India have tried to replicate others’ findings.

The open-ended responses were analyzed using content analysis as mentioned in the data analysis section. The extracted themes or categories from this open-ended question are “Insufficient details”, “Conflicting results”, “Affirmative results”, “Ethical concerns”, and “Hopeful progress”. All the details about these themes have been given below with quotations from the responses. Open-ended responses indicated that this difference may be attributed to differences in access to resources required for replication, e.g., funding, and computing. In fact, researchers in both countries reported similar challenges during replication attempts. The most frequent among these challenges were resource constraints, specifically, time and money.

#### Insufficient details

Respondents who reported that they could only partially replicate existing studies, most mentioned lack of adequate information provided in the paper as the primary reason. Participants emphasized the importance of effective documentation for enabling reproducibility, noting that the specific requirements of this documentation vary by domain. For example, one respondent noted the importance of documenting model hyperparameters.

I work in the area of applications of deep learning (DL) to IoT. Most of the times, I have observed that the performance results for the DL models are not really replicable. The reason could be the authors don’t share all the hyperparameters for model training. However, even after trying with a range of hyperparameter settings, we couldn’t replicate the results. This even happens for the A* conference papers. Hope with your findings and the corresponding publications, the researchers will start thinking about sharing the required information to reproduce the results. – respondent from India, engineering

In some cases, a successful reproduction or replication was achieved by contacting the study’s authors to fill in missing information, highlighting the importance of cooperation and collaboration.

*It went well, we had to contact the authors to get some details that were not available in the paper, but they were responsive. Our results were affirmative.* – respondent from USA, computer science

However, other respondents reported instances where authors did not respond to their inquiries. **Particularly for researchers in India, contacting a study’s original authors and receiving a response appears to be more challenging.** While India is increasingly engaged in international networks, the country still faces barriers in this regard, influenced by geopolitical and economic factors. These barriers may play a role in lack of scientific dialogue.

*Many times the code and data can be obtained from the researchers. However, sometimes they do not respond and do not make the data/code available either. That is quite frustrating really. However, sometimes the easy way out is just to implement ourselves, compare, and then report in the paper, mentioning the code/ data was unavailable.* – respondent from India, information science

#### Conflicting results

Even when all relevant information and artifacts were available, some researchers found themselves unable to successfully reproduce published findings, often obtaining conflicting results with those reported in this original work. This experience was pervasive among researchers surveyed in both countries.

*We had enough information to repeat the study, a computer design study which was published at a well-known conference, but we got conflicting results.* – respondent from USA, computer science

*Tried to simulate based on the details provided by the author in the paper, but failed many times to reproduce the results shown.* – respondent from India, electrical Engineering

#### Affirmative results

Optimistically, many of our participants reported successfully reproducing and replicating findings in the literature, and many of them went on to extend those findings in their own studies. This was particularly the case for our respondents from the USA; who reported having affirmative results when they replicated others’ work which is much higher compared the India. Very few researchers mentioned about the affirmative results from India. But overall the statistics highlight that it was not very easy for the Indian researchers. All these percentages are from the group that tried to replicate others’ work.

*Successful. The reproduction was affirmative. I have also had others repeat studies my group has conducted.* – respondent from USA, computer science

#### Ethical concerns

Concerns about the authenticity of the data and the validity of results were raised, with some participants encountering fake data or unclear methodologies that hinder reproducibility.

*Not sufficient information was available. Requested to provide raw data. But analyzing the data revealed that it is fake. And it was of no use. But the article was published in a good journal.* – respondent from India (Respondent did not provide a research discipline.)

#### Hopeful progress

Better community practices such as sharing codes and detailed project pages (e.g., on platforms like GitHub or PapersWithCode) have made reproducibility easier over time. Proper documentation and accessible data and code are key to successful reproduction. Respondents indicated that in the past, reproducing others’ work was significantly more challenging due to inadequate documentation and the absence of code and data sharing. However, the situation has considerably improved in step with the open science movement.

*Earlier years it was tough. Many times code was not public and contacting the authors was fruitless. Also, the project pages were not well documented. So things were tough that time. Now the environment has changed. As others in the community make code public and make dedicated project pages, it is easier to reproduce them.* – repondent from India, computer science

*In three tries the answers vary. Case1 (in 1986) was a very difficult and time-consuming process that reflected the low standards of research process control at the time. Case2 (in 2006) went much better and the outcome was much closer correspondence between the published results and my student’s replication of them. Case3 (in 2019) was an exact replication using the code the authors had posted on OSF.* – respondent from USA, sociology

Experiences with reproducibility are varied; while some have never attempted it, others have had different levels of success. There are reports of both complete failures and successes in reproducing results, reflecting a broad spectrum of challenges and outcomes in the field.

### Attitudes towards open science

More than half of respondents in both countries reported some awareness of the open science movement using a closed-ended question. Specifically, in the USA, 76.62% of respondents indicated awareness vs. 61.78% in India. In both countries, social science researchers were more familiar with the open science discourse than engineers (see Fig 1(c)). In India, 75.55% of respondents from social science reported they had heard about the open science movement; this statistic was 57.53% for engineers. Overall support for open science principles was assessed on a 7-point Likert scale, ranging from *no support* to *strong support* (see Fig 2). We observe most of the respondents support open science practices, and the percentage indicating strong support is similar for both countries.

#### Reasons not to support open science

There was an open-ended question in the survey for those respondents who do not support open science so that if they want to mention their reason for no support, they can. Some of the respondents who indicated no support or slight support for the open science movement mentioned their concerns as expected. After analyzing responses from Indian researchers we observe that open access fees are a significant barrier. Some also expressed concerns that research quality is lesser in paid journals, or that journals’ incentives may be skewed by this model. Overall, the benefits of open science were not always clear to them.

*Unless I know what it really is, guessing an answer here would be unrealistic. Is this the same as “Open Access”? If yes, I oppose it. This ’paid stuff’ has diluted science alike.* – respondent from India, electrical engineering

Relatedly, demands for open science may favor elite institutions and increase inequity.

*If I were to not wholeheartedly support it, it might be because open science movement is restricted to elite institutions, led by researchers from developed countries where the resources available and the challenges faced by the researchers are very different from the ones faced by researchers from the developing countries (participant unavailability, low incentives for participation in research, power failures, lack of lab space, non compliance of participants to protocol despite consent unique to developing countries, some institutes do not have ethics board to approve a study etc).* – respondent from India, psychology

*It shifts costs from wealthy publishers to scholars who can’t afford it and disproportionately harms junior scholars and those from poorer institutions so it exacerbates inequality across the board.* – respondent from USA, Political science

Respondents cited concerns about the time and cost associated with sharing data and code. Several respondents compared the sharing of resources to revealing proprietary work before completing a long-term project which can increase their competition. They shared they could write multiple papers based on a single dataset or piece of code, and that they were reluctant to share with others to avoid heightened competition.

*Some code takes years to produce and researchers are still publishing papers from it after one paper is published. Sharing it means that you are essentially giving away your proprietary work before a long-term project is complete.* – respondent from USA, sociology

*if we open everything then we take the risk to have more competition. That’s good to open but after a few years so that we can still publish our ideas before all the other people use our published work. If a lot of people have access to our last results and can replicate them, then they can easily work on the same topics (assuming they have more students in their labs)* – respondents from USA, engineering

Researchers engaged in human subjects and qualitative research expressed difficulty in making all aspects of their work openly accessible due to privacy concerns.

*Human subjects research requires sensitivity for confidentiality and privacy. In public health research there can be powerful opponents who may use data inappropriately. People untrained in statistics and epi could obfuscate important health issues - erodes respect for experts.* – respondent from USA, social science

One researcher from the USA suggested that the federal government should take the necessary steps for proper open science practices.

*I’d like there to be more open science, but federal grants are not willing to pay for it in any meaningful way. For example, the new OSTP memo on open access publishing and data was put out a year ago, no follow-up from OSTP, and now each federal agency is coming up with their own set of requirements based on their interpretation of the memo. It’s going to be chaos for folks who have funding from multiple agencies because now they’re all going to have different requirements AND none of them are going to pay for the time and effort required to comply with everything. I went to NASA’s webinar on their new policy and they had very vague answers to some pretty basic questions from faculty and scientists about their new policies - it was a mess. Either the federal government gets serious about open science or it doesn’t happen.* – respondent from USA, mechanical engineering

#### Attitudes towards preregistration

We explored participants’ perspectives on a core open science practice, namely, preregistration [62]. In this survey we have used the term pre-registration due to its close ties with the open science movement and replication crisis. There are other different terminologies related to this topic which is only “registration” or more precisely “prespecification”. Therefore the findings of this survey question are very terminology-specific. We asked the researchers have they ever pre-registered for a study or not with the options “yes”, “no”, and “other”. The other option was provided so that if they have any other thing in mind to mention. Experience with preregistration varies between India and the USA, with only 15.71% of researchers in India reporting preregistration compared to 34.86% in the USA. **In India, only 8.88% of social science researchers reported having ever preregistered a study, as opposed to 43.92% in the USA.** Notably, however, the percentage of engineering researchers in the USA who have preregistered work was lower at 11.11% than in India at 17.81% (see Fig 1e). The results underscore how disciplinary culture can be as meaningful, if not more so, than country-specific norms.

The responses from the “other” option suggest that the preregistration process is generally not clear to many researchers.

*I think so? I’ve filled out an IRB for a human subjects study - does that count?* – respondent from USA, mechanical engineering

It is noteworthy that while pre-registration is less common in engineering, the field still upholds rigorous scientific methods and practices.

*My work does not involve statistical work of the kind implied in this question. We are physical scientists so we don’t need pre-registration.* – respondent from India, mechanical engineering

One respondent pointed out that preregistration may not be relevant for most studies, further emphasizing varied perceptions among researchers about the utility of preregistration.

*Preregistration is stupid for the vast majority of studies. If you are doing a high-risk/cost intervention hypothesis test, fine. But very few people are doing that.* – respondent from USA, sociology

### Peer review to ensure reproducibility

We asked our participants whether they had ever been asked to share data files, code, or other materials during peer review of their own work. This question was open-ended and we used content analysis to analyze their responses. To explore the role of peer review in promoting reproducibility and replication, researchers were surveyed about their experiences with the peer review process. Specifically, they were asked if they had been requested to share data files, analytic code, or other research-related materials during the review. This inquiry was posed as an open-ended question, allowing participants to freely share their thoughts and experiences. Through content analysis of their responses, we identified six major themes: “Never”; “Sometimes”, “Rarely”; “Yes”; “Provided always”, and “If accepted”. We have counted the frequency of these categories/themes. The theme “Never” represents those responses who mentioned they never got any questions during peer review to share data/code or other materials, “Rarely” for those responses where the respondents mentioned they received it very few times, “Sometimes” represents moderate responses, “Yes” means they always receive responses to provide all these materials during peer review, “Provided always” represent those responses who mentioned they never received questions regarding material sharing because they always share/provided it, and “if accepted” represent those respondents where the researchers mentioned explicitly that they only asked to share if that paper got accepted for publication. (see Fig 1(f)).

*Many top computer vision or machine learning conferences now encourage code submission during paper submission. However, as a reviewer (due to time pressure) I am not willing to check the code by running it. So, I think, ultimately it comes down to reducing the load on the reviewers which, in turn, means training more reviewers to do the job right.* – respondent from India, engineering

Also, we found some other important key aspects. Some respondents mentioned that sharing data and code is part of the mandatory requirements for certain conferences or journals. Others mentioned that they share data and code upon request or via repositories. A few respondents highlighted that they use platforms like Anonymous GitHub to submit code or data during the review process and make it public after acceptance. Qualitative research or theoretical work often sees fewer questions on reproducibility. These all depend on the type of research, the journal, and conference policies.

### Institutional challenges and opportunities

As noted, our survey explored obstacles to successful reproduction and replication. These include the unavailability of code or data, unclear explanations of experimental settings, and lack of detailed methodological descriptions. Our survey protocol also allowed respondents to enter “other” factors they perceived to contribute to lack of reproducibility in their field. The majority of these “other” issues were institutional or systemic, related to research culture, norms, incentives, peer review processes, and training.

#### Misaligned incentives

The most commonly noted of these factors across both countries and both fields was **lack of incentives** for reproducing or replicating others work. Few journals regularly publish reproduction, but rather highlight novelty. This has substantial impact given respondents reporting feeling significant pressure to publish.

**Fig 4:**
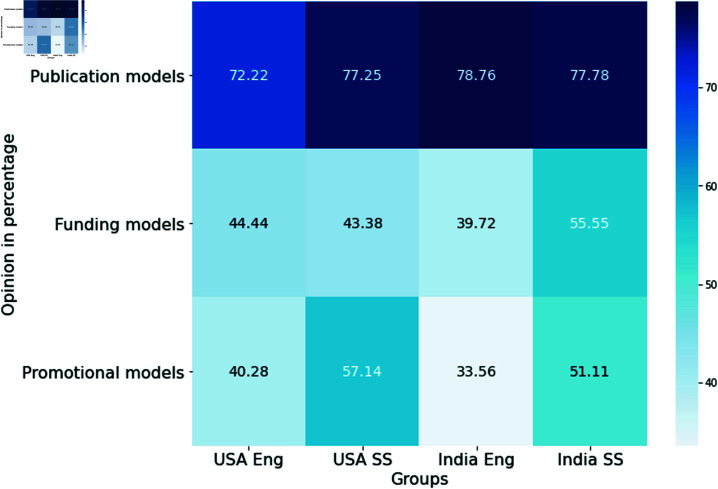
Changes to Models needed according to the participants.

We asked participants to choose from a range of institutional changes that could help support reproducible research practices, with the option to select multiple choices. Across both countries, the most commonly selected response was “changes to publication models” (78.53% of researchers from India and 75.86% from the USA). 57.14% social scientists from the USA suggested “changes to promotional models” are required to incentivize best practices. In both countries, engineering researchers reported “changes to funding models” to be equally or more important than changes to promotional models (see Fig 4).

We also provided an “other” option; using this option many respondents noted the important role of rigorous peer review. According to many, existing peer review processes are not sufficient to understand replicability and data sharing. Suggested changes to publication models include more rigorous peer review and mandatory sharing and review of code, materials, and data.

*Not pushing the idea that all novel work must be surprising or disconfirming previous folk understanding of an issue.* – respondent from USA, psychology

*Review and publication process should really be focusing on the rigor of the methods, not the significance of the results; with valid and generalizable methods, insignificant/unexpected results are still important, which means we thought it wrong.* – respondent from USA, psychology

#### Coursework

A hope of the open science movement is that a global shift toward more rigorous research practices can be achieved through educational efforts targeting the next generation of researchers. We asked participants about their willingness to incorporate reproducibility and open science topics into their coursework. In India, 57.78% of social science researchers and 47.94% from engineering believed it is necessary to add open science and replication topics to current course work. In the USA, these numbers were slightly lower; 46.03% of respondents from social science and 38.89% from engineering believed this is needed (see Fig 1(d)).

One researcher mentioned his concern is to fit this within current curricula.

*Yes, it would be great. But I’m not sure how we could fit it into the curriculum.* – respondent from USA, materials science

### Signals of credibility of published findings

We sought to understand how our participants evaluate the credibility of published findings when they see them in the literature, e.g., based on journal reputation, whether authors have shared materials, robustness of study design, and similar. We offered a lengthy list of potential signals (e.g., open data, open code, study design, theoretical basis, sample size, author reputation, journal reputation) and also left space for respondents to include their own, understanding that important signals likely vary across domains. Participants were allowed to select as many features as they deemed important.

**Fig 5:**
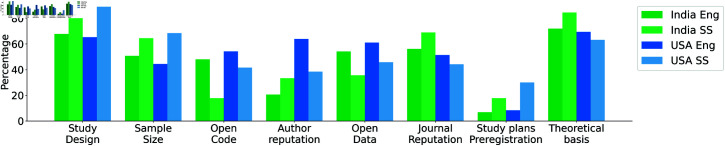
Signals of credibility.

Study design and theoretical basis were the most frequently identified across both countries as signals of credibility. Engineering researchers tend to look for open sharing of data and code, whereas social science researchers evaluate the sample size of the study population. Author reputation and journal reputation were also identified as relevant factors, particularly by respondents from the USA (see Fig 5).

*I typically pay extremely close attention to the detail with which the data and analysis are described. When authors are not careful in how they describe what they did, this is a major red flag. Obviously, careful description can mask uncareful data collection, design, and analysis, but it is still a signal of credibility that I look to.* – respondent from USA, political science

Other features mentioned by the respondents included knowledge of the literature, correspondence between theory and evidence, sensitivity checks, ideological biases, and plausibility of conclusions.

## Limitations

Our comparative analysis focuses on research cultures in India and the USA, across a set of social science and engineering disciplines. We note that the insights and conclusions drawn are specific to these two countries and disciplines and may not be generalized outside of these contexts. We acknowledge including researchers from Africa, China, or Russia could have introduced greater diversity in the findings. These regions have distinct academic and research traditions, and their infrastructures for research integrity may be in different stages of development. Including a wider range of countries from the Global South would likely offer a more comprehensive understanding of how research integrity issues are viewed and addressed across different contexts, particularly in regions where these conversations are just beginning. Future research could aim to include a more geographically diverse sample, incorporating regions like Africa and China, to explore how research integrity awareness varies. Another limitation of this study is the absence of preregistration, which helps to reduce potential biases and mitigates selective reporting by clearly outlining the research questions, hypotheses, and analysis plans before data collection begins. While a detailed protocol was followed throughout the study, this study does not have any hypothesis, it is exploratory in nature.

## Discussion and recommendations

Our study offers an in-depth analysis of researchers’ views on reproducibility, replicability, and open science practices in the USA and India. While Western nations have more proactively tackled the replication crisis, Indian researchers are becoming more aware of these issues and are making strides toward embracing open science. Our findings indicate that the adoption of open science and best practices face many common challenges, although researchers in India report additional hurdles. Here are the key takeaways summarized.

**Policies and incentives.** Respondents across contexts highlight the misalignment of prevailing academic incentives, centered around publications, promotions, and funding, as detrimental to engagement with open science practices. They note a lack of appreciation for replication studies in favor of novelty [63,64].

We note that new incentives have emerged and suggest that they should be used as stepping stones for substantial extension. One example is badges. These simple rewards have a real, measurable impact on engagement with open science practices [65,66]. Next steps might include the badges into reputational metrics and promotional practices. Pervasive implementation of badging will require resources, both human and computational.

Globally, establishing metrics to measure engagement with open science and replication efforts will be critical for incentive realignment. Once measurabe, these practices can be rewarded.

**Equity and bias.** Discussions of reproducibility and open science centered in the West have not fully appreciated the challenges faced by researchers working at institutions with fewer resources and less social capital. For example, when attempting to reproduce or replicate a published finding, respondents from India report significant challenges receiving responses from the paper’s authors as compared to those in the USA. While reproduction and replication should ideally be possible without consultation with authors, consultation is still standard practice and ultimately it is a biased practice that favors well-established community members.

Our work highlights the importance of economic factors as well. Wealthier institutions and regions can better afford open access fees [67]. This finding is consistent with prior work observing that the geographic diversity of authors is greater for non-open access articles than for open access articles [68].

**Education.** Our findings suggest the value of educational and public-facing initiatives in India, e.g., workshops, consortia, and centers, where questions about reproducibility and replication have simply received less attention. Yet, our findings also suggest that the mere existence of institutions aiming to improve research practices in India can not be successful without effective execution at the grassroots level. Comprehensive integration of training and access to resources is needed to facilitate the widespread adoption of open science practices. These resources include but are not limited to: time; computing resources; support for data storage and management; and funding for open-access publication fees.

*Coursework.* Our study suggests universities in both countries should have undergraduate and graduate courses focused on best practices in research. These might be full courses or well-designed course modules thoughtfully situated within existing curricula. At the moment, many universities have yet to implement any courses.

*Clarify the purpose and benefits of preregistration.* It was clear from responses that social science researchers in the USA were the only group widely committed to preregistration. Most researchers were not familiar with pre-registration and some had fundamental misunderstandings of the concept [69]. Successful progress toward reproducible and replicable science must increase awareness and understanding of what preregistration entails through workshops, seminars, and training sessions. Provide clear, step-by-step guidance and examples of well-executed preregistrations which could include standard templates or checklists that align with various types of studies.

**Rigorous peer review:** Our work highlights the urgency of reevaluating existing peer review processes. Ideally, the strongest signal of credibility for a published finding should be the fact that it was peer-reviewed and ultimately published. A majority of our respondents expressed dissatisfaction with current peer review processes and suggested that more rigorous mechanisms of research assessment are needed. Researchers expected to publish rigorous, reproducible, replicable work, adhering to best practices of open science are embedded within social, cultural, and economic contexts. Our work makes clear that this context is not uniform and that solutions that do not consider this will inevitably fall short.

## Conclusion

Our work offers insights and recommendations that we envision can inform a variety of research stakeholders, most notably institutions responsible for research policy and funding, universities, and publishers. We highlight the research climate in India to broaden this dialogue beyond its Western tradition and to understand the barriers encountered by Indian researchers on a day-to-day basis. Our findings suggest a need for global reassessment of the economic and reputational biases that currently influence the accessibility and implementation of open science. These biases exacerbate inequities, particularly affecting researchers from resource-limited settings and those early in their careers. Addressing these challenges requires a clear understanding of the diverse contexts in which researchers operate, ensuring that the move towards open science is inclusive and equitable.
